# Survey of Oxygen Delivery Practices in UK Paediatric Intensive Care Units

**DOI:** 10.1155/2016/6312970

**Published:** 2016-07-19

**Authors:** Sainath Raman, Samiran Ray, Mark J. Peters

**Affiliations:** Respiratory, Critical Care and Anaesthesia Group, Infection, Immunity, Inflammation and Physiological Medicine Programme, Institute of Child Health, University College London and Great Ormond Street Hospital, Great Ormond Street, London WC1N 3JH, UK

## Abstract

*Purpose.* Administration of supplemental oxygen is common in paediatric intensive care. We explored the current practice of oxygen administration using a case vignette in paediatric intensive care units (PICU) in the united kingdom.* Methods.* We conducted an online survey of Paediatric Intensive Care Society members in the UK. The survey outlined a clinical scenario followed by questions on oxygenation targets for 5 common diagnoses seen in critically ill children.* Results.* Fifty-three paediatric intensive care unit members from 10 institutions completed the survey. In a child with moderate ventilatory requirements, 21 respondents (42%) did not follow arterial partial pressure of oxygen (PaO_2_) targets. In acute respiratory distress syndrome, cardiac arrest, and sepsis, there was a trend to aim for lower PaO_2_ as the fraction of inspired oxygen (FiO_2_) increased. Conversely, in traumatic brain injury and pulmonary hypertension, respondents aimed for normal PaO_2_ even as the FiO_2_ increased.* Conclusions.* In this sample of clinicians PaO_2_ targets were not commonly used. Clinicians target lower PaO_2_ as FiO_2_ increases in acute respiratory distress syndrome, cardiac arrest, and sepsis whilst targeting normal range irrespective of FiO_2_ in traumatic brain injury and pulmonary hypertension.

## 1. Introduction

The administration of supplemental oxygen is common in the critically ill. The aim is to augment oxygen delivery to the tissues [1]. Hyperoxia can lead to production of reactive oxygen species and cell injury [2, 3]. Conversely, hypoxia causes cell death. The “ideal” PaO_2_ target range is unclear. Consequently clinical practice varies.

Eastwood et al. reported that 77% of intensivists in Australia and New Zealand prescribed oxygen saturation targets. Clinicians working in regional centers were less concerned with oxygen toxicity [4]. De Graaff et al. explored the response of Dutch clinicians to arterial blood gas values (ABG) in tertiary intensive care units. The FiO_2_ was reduced in only 25% of situations with a PaO_2_ > 16 kPa [5].

The etiology and evolution of paediatric critical illness are different to adults. Multiorgan failure (MOF) occurs early in children and they have better survival [6, 7]. Nonetheless, the duration of mechanical ventilation and length of stay in the paediatric intensive care unit is increasing [8]. This survey aimed to describe prevalent paediatric intensive care practice, existence of weaning protocols, and if a clinical equipoise exists between liberal and restrictive oxygenation targets.

## 2. Material and Methods

All the members of the Paediatric Intensive Care Society (PICS), UK, were requested to complete an online survey. PICS consists of nursing, medical, and allied health professionals working in paediatric intensive care units. The practitioners from the neonatal intensive care units in UK were not approached, as their patient profile is significantly different.

The survey was designed by the authors and published using a survey website (https://opinio.ucl.ac.uk). Demographic data including age, ICU type, their seniority, and years of practice were sought. The study was discussed with the chair of Bloomsbury Research and Ethics Committee (London, UK). We were advised that a formal ethics review was not required.

The survey outlined the following clinical scenario: a 1-year-old patient with no premorbid conditions is ventilated with peak inspiratory pressure of 28 cm H_2_O, positive end expiratory pressure of 6 cm H_2_O, respiratory rate of 20 breaths per min, and FiO_2_ of 0.8. His peripheral oxygen saturation (pulse oximetry), heart rate, blood pressure, and mean blood pressure are 94%, 125 beats per min, 85/56 mmHg, and 66 mmHg, respectively. He has bilaterally equal and reactive pupils measuring 3 mm. He is sedated on intravenous morphine and midazolam. He is not paralysed. Latest arterial blood gas values are as follows: pH: 7.32, PCO_2_: 6.2 kPa, PaO_2_: 10 kPa, BE: -ve 4, and lactate: 1.5 mmol/L. The PIM2 predicted risk of mortality is 8.8%. He has been ventilated for 2 days.

With the same clinical history, clinicians were asked to decide on the oxygenation targets when the potential diagnosis is ARDS, CA, Sepsis, TBI, or PHTN.

A further question explored if weaning protocols were in place in their units. The need for a randomised control trial (RCT) with tight arterial oxygenation targets was explored.

## 3. Results

Only 30% (53) of those whom were invited to participate in the online survey responded. The majority of respondents worked in moderate sized ICUs, with admission rates between 500 and 1000 patients per annum. The characteristics of the respondents are presented in Table 1.

The majority of units (96%) had an alarm target on their oxygen saturation monitor. Thirty-eight respondents (73%) worked in units that did not have an oxygen weaning protocol for mechanically ventilated patients. The units with admissions more than 1500 were less likely to have a weaning protocol compared to those between 500 and 1500 admissions.

For the given clinical scenario, 21 respondents (42%) did not follow PaO_2_ targets. Of the rest, 21 clinicians (42%) targeted PaO_2_ between 8.1 and 10 kPa. Only 8 (16%) aimed for the normal range (10.1–13 kPa).

**Figure 1:**
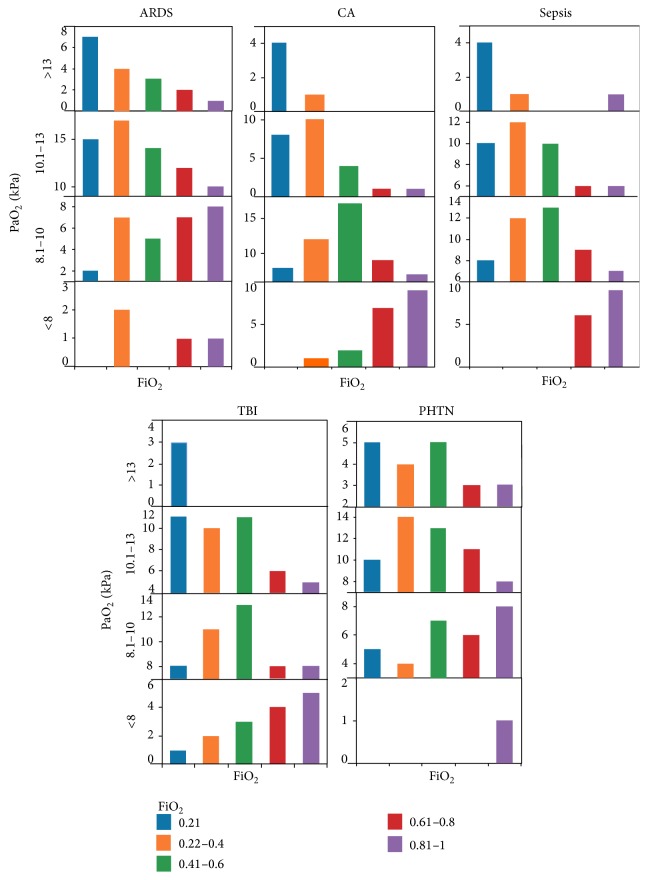
The profile of PaO_2_ (*y*-axis) and FiO_2_ (*x*-axis) targeted in 5 clinical scenarios in a child with moderate ventilatory requirements. The PaO_2_ ranges from <8 kPa in the bottom panel to >13 kPa in the top panel within each scenario. FiO_2_ ranges from 0.21 (blue) through to 0.81–1 (purple). The three scenarios in the upper section show a pattern of more restrictive PaO_2_ targets with increasing FiO_2_. The 2 scenarios in the lower section show that higher normal PaO_2_ ranges are targeted irrespective of increasing FiO_2_.

In ARDS, CA, and sepsis, there was a tendency to aim for lower PaO_2_ (<10 kPa) as the FiO_2_ increased. This was noticeable when the FiO_2_ was more than 0.4 (45%) which equates to a PaO_2_/FiO_2_ ratio of less than 200. Following TBI and in PHTN, there was a propensity to aim for normal PaO_2_ (10.1–13 kPa) even as the FiO_2_ rose (28–33% when FiO_2_ > 0.4). In TBI, the proportion of respondents targeting a lower PaO_2_ increased when the FiO_2_ was more than 0.8 (8%). A proportion of respondents targeted PaO_2_ ranges above normal (15%). In PHTN, normal range remained the preferred range throughout the range of FiO_2_ (Figure 1).

The initial scenario was further extended as “no improvement after 24 hours of intensive care.” The management strategy did not change in this setting.

Thirty-nine percent considered it ethical to conduct a RCT with tight arterial oxygenation target whilst 11% did not. The remaining respondents were undecided.

## 4. Discussion

Our survey shows that, practice variation notwithstanding, there seems to be a general consensus to aim for lower PaO_2_ in the setting of ARDS, CA, and sepsis. The results are consistent with higher PaO_2_ targets being chosen in children following TBI and in PHTN. Only a small proportion of respondents felt a RCT with tight oxygenation target would be unethical.

Paediatric intensivists tolerate a low SpO_2_ target (88%) with a low tidal volume strategy for ARDS [9]. Our findings concur. A recent point prevalence study reported that adult intensive care practitioners aim to prevent low oxygen saturation (SpO_2_ < 90%) but fail to address high saturations [10]. This is in the face of mounting evidence of harm from hyperoxia [11]. Should we aim for a restrictive oxygenation target in critically ill patients? The “HOT or NOT” trial showed that separation between titrated oxygen target and standard target is possible in intensive care [12]. A recent multicenter study demonstrated no difference in 90-day mortality between mechanically ventilated patients randomised to a conservative (pulse oximetry: 88–92%) and liberal oxygenation targets (>96%) [13]. A larger randomised control trial is awaited.

The main limitation of this survey is the likely low response rate. At the time of the survey the membership of the society was not well defined. Responses were not sought beyond a single e-mail. The low number of respondents from junior staff perhaps suggests that considerable experience is needed to set distinct targets in these clinical scenarios. Despite this limitation, the results indicate that restrictive targets are aimed for in certain scenarios.

We had intended to analyse Cohen's kappa to look at interrater agreement. However, due to the small sample size a formal statistical analysis was not attempted.

## 5. Conclusions

In this study variability and lack of consensus are consistent with an assumption of clinical equipoise. Supplemental oxygen administration practices and oxygenation target practices vary. A majority of respondents worked in units with no oxygen weaning protocol. A proportion of clinicians do not follow PaO_2_ targets. Clinicians aim for lower PaO_2_ thresholds in ARDS, CA, and sepsis whilst aiming for the normal range in TBI and PHTN. The lack of consensus and the large variability in practice demonstrate equipoise. This should be addressed with a feasibility trial comparing restrictive to standard oxygenation targets in critically ill children to lead up to a future RCT.

## Figures and Tables

**Table 1:** Characteristics of respondents.

Number of admissions/year to your intensive care unit	Number (%)
<500	9 (17.6)
501–1000	24 (47)
1001–1500	13 (25.5)
>1500	5 (9.8)
No response	2

Cardiosurgical center	33 (66)

Neurosurgical center	35 (67)

Grade of respondent	

Consultant	25 (48)
Senior nurse	10 (19.2)
Senior fellow	12 (23)
Junior fellow	5 (9.6)
No response	1

Number of years of practice in intensive care	

2–5 years	13 (25.5)
5-6 years	13 (25.5)
>10 years	25 (49)
No response	2
